# Giant true hepatic aneurysm mimicking Mirizzi syndrome

**DOI:** 10.1016/j.jvscit.2020.09.009

**Published:** 2020-09-25

**Authors:** Christine L.S. Corion, Patrick W.H.E. Vriens, Ian P.J. Alwayn, Jaap F. Hamming, Jan van Schaik

**Affiliations:** a Department of Surgery, Leiden University Medical Center, Leiden; b Department of Surgery, Elizabeth-TweeSteden Hospital, Tilburg

**Keywords:** Visceral aneurysms, Mirizzi syndrome, Giant hepatic artery aneurysm, Open vascular reconstruction

## Abstract

Giant true aneurysms of the hepatic arteries are rare. Pseudoaneurysms of the hepatic arteries are more common and are mostly caused by intra-abdominal infection, iatrogenic injury, or trauma. Hepatic or cystic pseudoaneurysms are often successfully treated by embolization owing to their saccular nature as opposed to true aneurysms. We present a case of a patient with a giant true aneurysm of the proper hepatic artery, mimicking Mirizzi syndrome. Open reconstruction was successfully preformed, and the patient made a full recovery.

Giant aneurysmal disease of the hepatic artery is not uncommon. It usually concerns pseudoaneurysms after trauma, intra-abdominal infection, or invasive diagnostic and therapeutic treatment.^1^^,^^2^ Giant true hepatic aneurysms, defined as exceeding 5 cm in diameter, are rare.^3^, ^4^, ^5^ Mirizzi syndrome is defined as common hepatic duct obstruction by compression from an impacted gallbladder stone.^6^^,^^7^ Symptoms may consist of jaundice, fever, and right upper quadrant abdominal pain.^8^ Patients in whom Mirizzi syndrome is not recognized preoperatively have a higher risk of morbidity and bile duct injury during surgery.^9^^,^^10^ We present a rare case of external compression of the common bile duct by a true aneurysm of the hepatic artery mimicking Mirizzi syndrome. Consent was obtained from the patient for publication of this case report.

## Case report

A 72-year-old Caucasian man was referred for evaluation of jaundice. He had been experiencing postural abdominal pain for more than 1 year. In the past month, the pain was getting worse and he experienced heartburn. He noticed loss of appetite in the past 2 months and lost 8 pounds of bodyweight. During the last weeks, jaundice developed, his urine became dark, and he began having gray-colored stool in the absence of fever, chills, or vomiting. There was no history of hepatitis, nor recent travel outside of the Netherlands. Previous medical history included a transient ischemic attack in 2010. He stopped smoking 15 years ago. Up to 2 months ago he drank 3 glasses of wine a day. Medication included a statin and dual antiplatelet therapy, none of which is known to cause jaundice as a possible side effect.

An outpatient ultrasound examination showed a large aneurysm of 10 cm central in the liver hilum; there were no gallstones. On admission, his vital signs were normal. Physical examination revealed an abdominal mass with an audible murmur in the epigastric region. There were no signs of hepatomegaly. Laboratory findings showed elevated levels of total bilirubin (114 μmol/L), aspartate aminotransferase (AST; 198 U/L), alanine aminotransferase (ALT; 472 U/L), AF (1508 U/L), gamma-glutamyl transferase (1742 U/L), C-reactive protein (33.9 mg/L), and normal white blood count (8.5 10^9^/L).

A triple-phased contrast-enhanced computed tomography (CTa) scan revealed a true aneurysm starting in the distal common hepatic artery, reaching up to the bifurcation of the proper hepatic artery of almost 13 cm, causing compression of the common bile duct with intrahepatic bile duct dilatation (Fig, *A* and *B*). Also, a small subthreshold infrarenal aortic aneurysm of 5.1 cm was seen. No other intra-abdominal abnormalities were found. Semiacute reconstruction of the hepatic artery was performed. A bypass was created from the common hepatic artery to the bifurcation of the proper hepatic artery, using a reversed autologous saphenous vein graft. Clamping time to the liver was approximately 45 minutes. Arterial flow in the right and left hepatic arteries was preserved, but the gastroduodenal artery had to be sacrificed owing to dissection and subsequent thrombosis.

**Fig:**
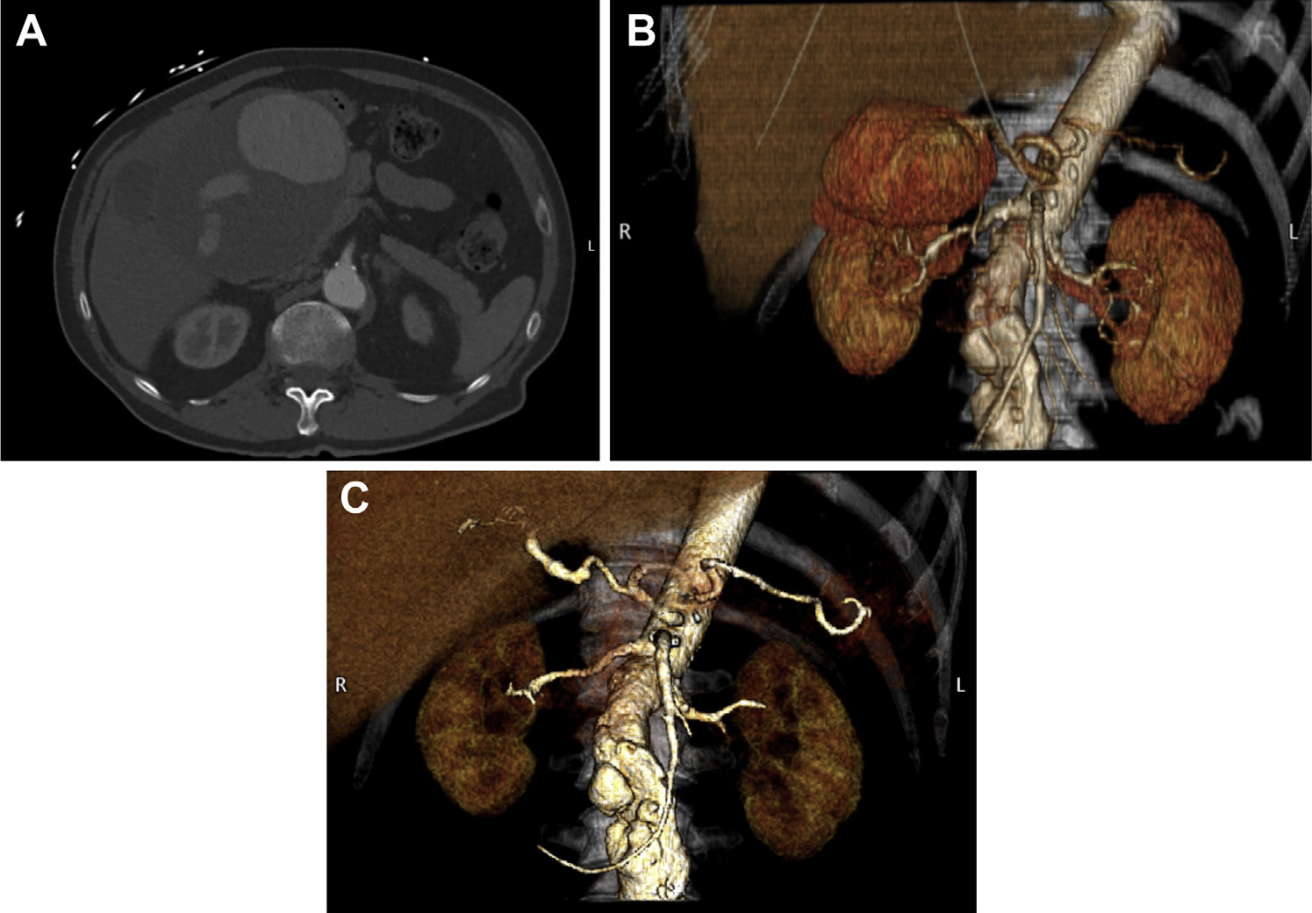
**A,** Abdominal computed tomography (CTa)scan showing intact giant true hepatic artery aneurysm compressing the liver hilum. **B,** Three-dimensional reconstruction showing the hepatic artery aneurysm. **C,** Three-dimensional reconstruction after repair.

The total blood loss was 1.5 L; there were no perioperative complications. One day after surgery the total bilirubin level decreased to 50 U/L and the jaundice resolved. AST and ALT levels improved direct after the surgery (AST 109 U/L; ALT 266 U/L), suggesting no significant ischemia of the liver had occurred. He was discharged on postoperative day 5 and further recovery was uneventful. Duplex ultrasound examination 1 week after discharge and a CTa scan 4 weeks after discharge showed a patent reconstruction without complications (Fig, *C*).

## Discussion

We performed a systematic literature search for visceral aneurysms causing Mirizzi syndrome (Table). Forty articles were identified, and 15 were deemed eligible for our study. Articles only describing Mirizzi syndrome or visceral aneurysm treatment were excluded. The vast majority of reported hepatic artery aneurysms were false or pseudoaneurysms caused by abdominal trauma, previous hepatobiliary surgery such as orthotopic liver transplantation, or other interventions such as percutaneous liver biopsy. There is also an association with abdominal sepsis and other inflammatory processes such as pancreatitis.^1^^,^^2^ Giant true hepatic artery aneurysms are rare. Our literature search yielded only three confirmed cases.^3^^,^^5^^,^^11^ These aneurysms were considerably smaller (<10 cm) and in only one case it caused bile duct compression. Moreover, one patient remained untreated owing to progressive malignancy, while in the other cases treatment is not discussed. Whether the etiology of true aneurysms is mainly atherosclerosis, or whether it is more frequently associated with connective tissue disorders or large vessel vasculitis is unknown.

**Table:** Review of the literature on aneurysms causing Mirizzi syndrome

Authors	Title	Type of aneurysm	Cause of aneurysm	Management
Anderson et al^1^	Mirizzi syndrome associated with hepatic artery pseudoaneurysm: a case report	Pseudoaneurysm of hepatic artery	Inflammation	Embolization
Sharma et al^12^	EUS-guided thrombin injection of cystic artery pseudoaneurysm leading to Mirizzi's syndrome and hemobilia	Pseudoaneurysm of cystic artery	Inflammation	Embolization
Anwar et al^13^	Hepatic artery pseudoaneurysm mimicking Mirizzi syndrome	Pseudoaneurysm of hepatic artery	Inflammation	Embolization
Lin et al^14^	Hepatic artery pseudoaneurysm presenting with Mirizzi syndrome and hemobilia	Pseudoaneurysm of hepatic artery	Inflammation	Embolization
Suzuki et al^15^	Unruptured cystic artery pseudoaneurysm accompanied by Mirizzi syndrome: a report of a case	Pseudoaneurysm of cystic artery	Inflammation	Embolization
Luu et al^16^	Unusual complications of gallstones	Pseudoaneurysm	Gallstones	Embolization
Parathithasan et al^17^	Cystic artery pseudoaneurysm: over warfarinisation and Management	Pseudoaneurysm of cystic artery	Inflammation	Embolization
England et al^18^	Endoscopic management of Mirizzi's syndrome	Pseudoaneurysm of cystic artery	Inflammation	Embolization
Nelsen et al^19^	Hemobilia and Mirizzi syndrome: a rare Combination	Pseudoaneurysm of cystic artery	Not specified	Embolization
Tirumani et al^20^	Imaging of the porta hepatis: spectrum of disease	No aneurysm	Not specified	Embolization
Fujimoto et al^21^	Ruptured cystic artery pseudoaneurysm successfully treated with urgent cholecystectomy: a case report and literature review	Pseudoaneurysm of cystic artery	Inflammation	Surgery
Fernandes et al^22^	Traumatic common hepatic artery injury causing isolated right hepatic ischemia due to a left accessory artery. A case report	Pseudoaneurysm hepatic artery	Trauma	Embolization
Raithel et al^11^	A true vascular aneurysm of the hepatic artery proper as a rare cause of nonmalignant painless jaundice	True aneurysm hepatic artery	Not specified	None
Marinis et al^23^	Vascular complications of large gallstones: Proposal of α first CLASSIFICATION	Pseudoaneurysm	Inflammation	Embolization/surgery
Julianov et al*^5^*	Hepatic artery aneurysm causing obstructive jaundice	Hepatic aneurysm	Not specified	None (patient wish)

*EUS*, Endoscopic ultrasound.

Our patient had no known risk factors for developing a false hepatic artery aneurysm. Although signs of atherosclerosis were present on the abdominal CTa, it seems to be mild and compatible with age. There was no evidence of connective tissue disorders or vasculitis-related disease. As in abdominal aortic aneurysms, the condition is rarely associated with symptoms. Only in the case of rupture or, as in our case, when compression of the common bile duct occurs, do symptoms develop. Our literature search revealed that hepatic or cystic pseudoaneurysms are often successfully treated by embolization. This finding can be explained by the fact that pseudoaneurysms are usually saccular in shape. Because the hepatobiliary vascular tree readily branches into important tributaries, endovascular treatment would impose a risk of occluding a right or left hepatic artery in true hepatic aneurysms, which are usually fusiform in shape. Open reconstruction is therefore the treatment of choice and seems to be feasible and safe. Although very rare, in jaundice a Mirizzi-like mechanism caused by a visceral aneurysm should be considered. If so, open vascular reconstruction seems the treatment of choice, because although embolization might prevent rupture, it would not relieve bile duct compression.

## Conclusions

Although very rare, in jaundice a Mirizzi-like mechanism caused by a visceral aneurysm should be considered. Open vascular reconstruction seems the treatment of choice.
